# Integrative multi-omics analysis implicates the RNF40-LIMA1 axis in hepatocellular carcinoma progression and immune microenvironment remodeling

**DOI:** 10.3389/fonc.2026.1846606

**Published:** 2026-06-29

**Authors:** Zhan Liu, Shaobo Wu, Kexin Fan, Chenhong Zhou, Yifei Liu, Jingyuan Xu, Yihan Liang, Saimire Wushuer, Xiaohan Song, Chen Cheng, Yaqun Guan, Yilei Zhang

**Affiliations:** 1 School of Basic Medical Sciences, Xinjiang Second Medical College, Karamay, Xinjiang, China; 2 The Institute of Molecular and Translational Medicine, Department of Biochemistry and Molecular Biology, School of Basic Medical Sciences, Xi’an Jiaotong University, Xi’an, China; 3 Beijing Normal University Karamay Affiliated Middle School, Karamay, Xinjiang, China; 4 School of Basic Medical Sciences, Xinjiang Medical University, Urumqi, Xinjiang, China

**Keywords:** hepatocellular carcinoma, LIMA1, RNF40, spatial transcriptomics, tumor microenvironment, ubiquitination

## Abstract

**Background:**

Ring Finger Protein 40 (RNF40), an E3 ubiquitin ligase, plays context-dependent roles in various cancers, serving as either a tumor suppressor or an oncogene. Critically, RNF40 targets LIM domain and actin-binding protein 1 (LIMA1) for degradation via the ubiquitin-proteasome pathway. LIMA1, which is involved in actin cytoskeleton organization, exerts opposing effects in different cancer types. However, the precise functional relationship between RNF40 and LIMA1 in Hepatocellular Carcinoma (HCC) remains poorly understood. Despite the efficacy of immune checkpoint inhibitors, intrinsic resistance affects 40-60% of patients, necessitating novel epigenetic and ubiquitin-related therapeutic targets.

**Methods:**

This study investigated the molecular mechanisms through which RNF40-mediated ubiquitination of LIMA1 influences Hepatocellular Carcinoma (HCC) progression. We utilized an integrative approach combining multi-omics analysis including bulk transcriptomics, single-cell RNA sequencing (scRNA-seq), and spatial transcriptomics with experimental validation using *in vitro* assays and *in vivo* mouse models. Differential expression analysis, survival outcome correlation, immune infiltration assessment, and cell-cell communication analyses were performed to characterize the roles of RNF40 and LIMA1 in HCC.

**Results:**

LIMA1 was significantly overexpressed in HCC across multiple transcriptomic and proteomic datasets (log2FC>1.5, *P* < 0.001), and its upregulation correlated with accelerated tumor proliferation, migration, invasion as well as an immunosuppressive checkpoint-enriched TME featured by M2 macrophage infiltration (R = 0.498, *P* < 0.001) and CD8+ T cell exclusion. Single-cell and spatial transcriptomic analyses indicated predominant LIMA1 enrichment in malignant cells and tumor lesions. LIMA1 overexpression facilitated HCC clonogenicity, migration, invasion and xenograft tumor growth. Conversely, ectopic RNF40 expression suppressed these malignant phenotypes, and such inhibitory effects were fully abolished upon concurrent LIMA1 overexpression both *in vitro* and *in vivo*. Re-analysis of TCGA-LIHC revealed that LIMA1 failed to serve as an independent prognostic marker after adjusting for clinicopathological covariates, warranting cautious interpretation of its prognostic implication.

**Conclusion:**

Our findings support the biological relevance of a functional RNF40-LIMA1 axis in HCC progression. Together with previously established biochemical evidence of RNF40-mediated LIMA1 ubiquitination and proteasome-dependent degradation, this study provides a rationale for further investigation of the RNF40-LIMA1 axis in HCC. The prognostic and immunotherapy-predictive value of LIMA1 requires validation in independent clinical cohorts and larger ICI-treated HCC datasets.

## Introduction

Hepatocellular carcinoma (HCC), a primary malignancy of the liver, ranks among the most common cancers worldwide, with over 900,000 new cases diagnosed annually (1, 2). As the fourth leading cause of cancer-related deaths globally, HCC imposes a substantial public health burden, exhibiting a consistently poor 5-year overall survival rate below 30% (2). Chronic hepatitis B (HBV) or hepatitis C (HCV) infection constitutes the primary risk factor, driving hepatocarcinogenesis through persistent inflammation and cirrhosis (3). Other established risk factors include metabolic disorders (e.g., obesity, diabetes), excessive alcohol consumption, and genetic predispositions (3). Despite therapeutic advances, late-stage diagnosis remains a critical challenge, contributing to the high proportion of advanced HCC cases (4). While targeted therapies such as sorafenib and lenvatinib serve as first-line treatments for advanced HCC, their efficacy is limited by the disease’s complex pathogenesis and frequent resistance to single-agent regimens (5). Consequently, identifying novel therapeutic targets, elucidating mechanisms of tumor progression, and expanding treatment options remain urgent priorities.

Actin-binding proteins orchestrate the assembly of actin monomers into dynamic cytoskeletal structures, enabling essential cellular processes including polarity establishment, adhesion, migration, division, and intracellular transport (6–8). Among these, LIMA1 (LIM domain and actin-binding protein 1) plays a pivotal role in cytoskeletal regulation. Notably, LIMA1 deficiency disrupts adhesion-catenin complex integrity, redistributes cadherin-catenin components (9, 10), and induces significant remodeling of the actin cytoskeleton that impairs cancer cell migratory capacity (11–14). However, the functional role of LIMA1 in HCC remains poorly characterized. Although our previous companion study (30) established RNF40 as an E3 ubiquitin ligase that promotes LIMA1 ubiquitination and proteasome-dependent degradation, whether the RNF40-LIMA1 axis contributes to HCC progression and tumor microenvironment remodeling remains unclear. In the present study, we integrated bulk transcriptomics, proteomics, single-cell RNA sequencing, spatial transcriptomics, and functional experiments to investigate the clinical and biological relevance of LIMA1 and RNF40 in HCC. A flow chart of the study can be found in **Supplementary Figure 1**.

## Results

### LIMA1 is overexpressed in HCC and shows heterogeneous prognostic associations across cohorts

**Figure 1:**
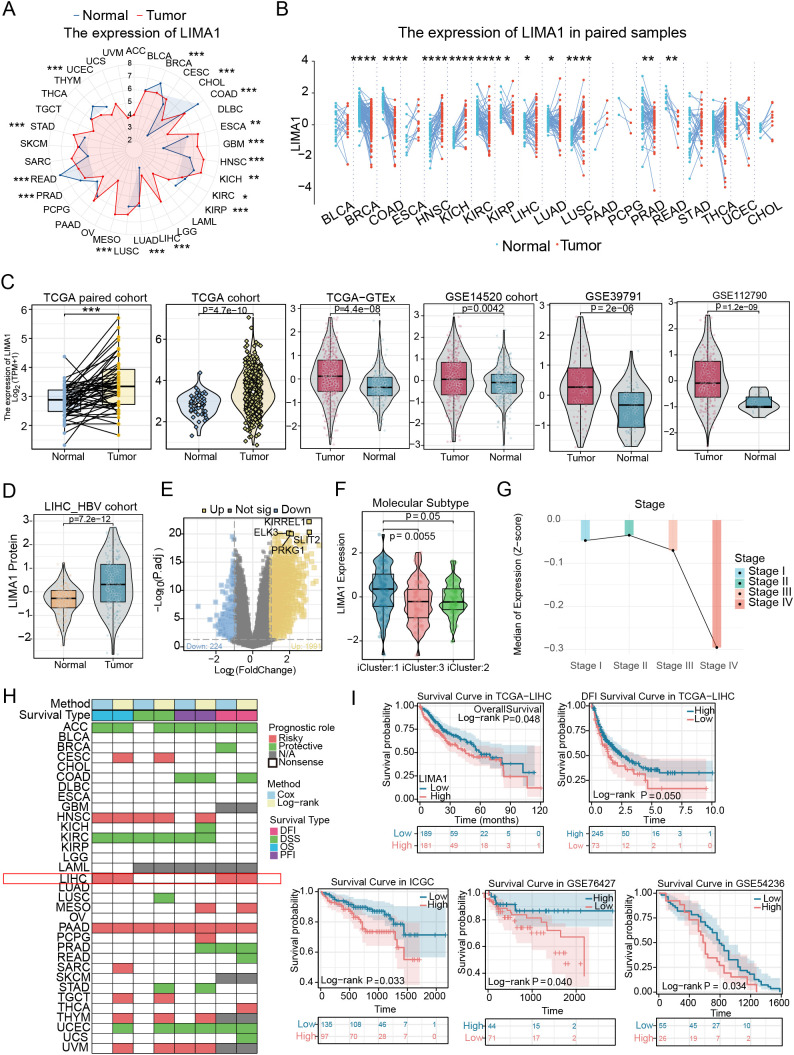
LIMA1 is overexpressed in HCC and shows clinical relevance. **(A)** Pan-cancer analysis of LIMA1 mRNA expression in tumor versus normal tissues. **(B)** Comparative LIMA1 expression in paired HCC tumor and adjacent non-tumor tissues. **(C)** Validation of LIMA1 expression patterns across multiple cohorts. **(D)** Differential protein expression analysis of LIMA1 in HCC tissues. **(E)** Volcano plot of differentially expressed genes between high- and low-LIMA1 expression groups**. (F-G)** LIMA1 expression across HCC molecular subtypes and clinical stages. **(H)** Kaplan-Meier curves demonstrating the association between LIMA1 expression and patient survival (overall survival, OS and disease-specific survival, DSS). Hazard ratios (HR) with 95% confidence intervals are shown. **(I)** Kaplan-Meier survival analysis of OS and DFI according to LIMA1 expression groups in TCGA-LIHC. Sample size, event number, cutoff method, hazard ratio, and log-rank P-value are indicated where applicable. *P < 0.05, **P < 0.01, ***P < 0.001, ****P < 0.0001.

Using the GEPIA database, we performed a pan-cancer differential analysis of LIMA1, which revealed significant and aberrant upregulation or downregulation of its expression across multiple cancer types (**Figures 1A, B**). To explore LIMA1’s potential role in carcinogenesis, we analyzed its mRNA expression in hepatocellular carcinoma (HCC) using expression profiles from the TCGA, GSE39791, GSE14520, and GSE112790 datasets.

Our analysis of HCC samples from the TCGA database showed a substantial increase in LIMA1 expression compared to normal tissue (**Figure 1C**). Paired differential analysis using samples from the same patients further confirmed that LIMA1 expression was significantly higher in HCC tissues than in adjacent normal tissues (**Figure 1C**). Subsequent evaluation of LIMA1 expression in independent GEO cohorts and the TCGA-GTEx cohort consistently demonstrated elevated levels in HCC tissues (**Figure 1C**). These findings indicate that LIMA1 is markedly overexpressed in HCC, suggesting its potential involvement in HCC development. Proteomic analysis further supported these results, revealing a significant upregulation of LIMA1 in HCC (**Figure 1D**). In the TCGA cohort, differential expression analysis between high- and low-LIMA1 groups identified 205 downregulated and 1,974 upregulated genes (**Figure 1E**). Given LIMA1’s overexpression, we next investigated its clinical relevance in HCC. Clinical correlation analysis revealed associations between LIMA1 expression and molecular subtypes as well as disease stage (**Figures 1F, G**). To assess the impact of LIMA1 expression on overall survival risk, we employed Cox proportional hazards modeling, which indicated that LIMA1 serves as a risk factor for HCC patients, with those in the high-expression group exhibiting poorer prognoses (**Figure 1H**). Survival curves for overall survival (OS) and disease-free interval (DFI) in the TCGA-LIHC cohort showed that HCC patients with elevated LIMA1 expression had significantly worse survival outcomes compared to those with lower expression levels (OS, P = 0.048; DFI, P = 0.05) (**Figure 1I**). Given the consistent overexpression of LIMA1 in HCC, we next evaluated its clinical relevance. Initial survival analyses suggested that LIMA1 expression may be associated with patient outcomes in selected datasets. However, after applying a consistent median cutoff in TCGA-LIHC, LIMA1 alone did not significantly stratify OS. In multivariable Cox regression adjusted for available clinicopathological variables, LIMA1 did not remain an independent prognostic factor. These results indicate that the prognostic relevance of LIMA1 is heterogeneous across cohorts and should be interpreted cautiously. Therefore, we considered LIMA1 as a candidate molecule associated with HCC biology rather than as a validated independent prognostic biomarker (**Supplementary Figure 4**).

This association with poor prognosis was also observed in the ICGC cohort, specifically in the GSE76427 and GSE54236 datasets. The difference in sample sizes between OS and DFI analyses reflects the availability of follow-up data, as 55 patients lacked DFI annotation due to incomplete recurrence follow-up or non-surgical treatment. Notably, in the GSE76427 cohort, the prognostic association followed a similar trend but did not reach statistical significance, which may be attributed to the smaller sample size (n = 115), differences in patient demographics (predominantly HBV-associated HCC in an Asian population), and technical variation between microarray (GSE76427) and RNA-seq (TCGA) platforms. These well-documented sources of inter-cohort heterogeneity are consistent with published literature on prognostic biomarker validation in HCC. We also analyzed RNF40 expression and the transcriptomic correlation between RNF40 and LIMA1 in TCGA-LIHC. RNF40-LIMA1 mRNA correlation was statistically significant but modest, supporting its use as transcriptomic context rather than direct evidence of post-translational regulation (**Supplementary Figure 5**).

### Single-cell and spatial transcriptomic analyses suggest tumor-enriched LIMA1 expression in HCC

**Figure 2:**
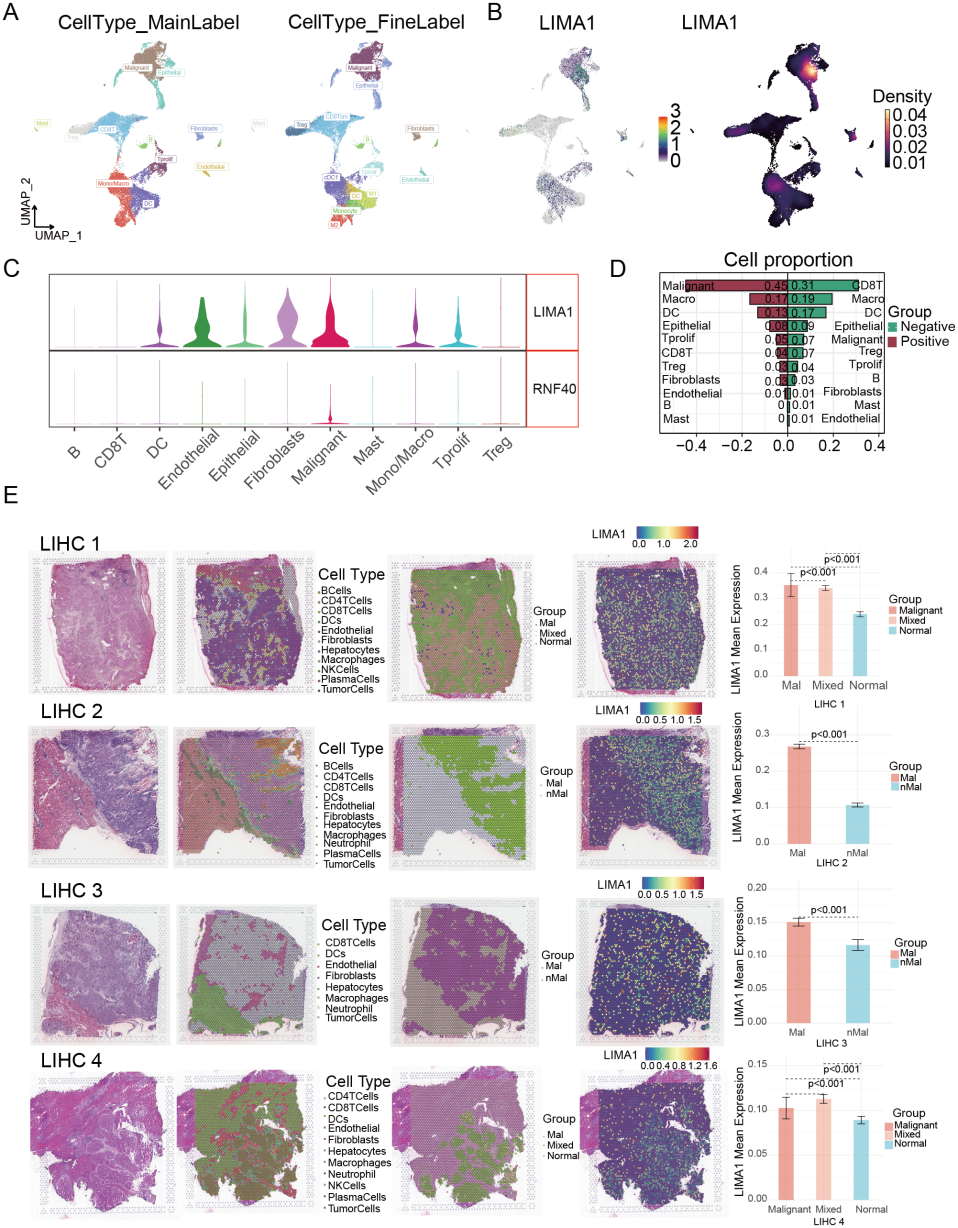
Single-cell and spatial transcriptomic analyses suggest tumor-enriched LIMA1 expression in HCC. **(A)** UMAP plot of broad cell clusters and major cell clusters. **(B)** Density plot showing the distribution of LIMA1 expression levels across cell types. **(C)** The expression distribution of LIMA1 and RNF40 in HCC cell clusters. **(D)** The proportion of each cell clusters in the LIMA1 positive/negative group. **(E)** The distribution of spatial tumor micro-environment and LIMA1 expression.

Single-Cell Transcriptomics Analysis, following rigorous quality control, batch effect correction, and standardization procedures, our single-cell transcriptomic analysis of two HCC patients revealed distinct cellular clustering patterns via UMAP visualization. The dataset was systematically classified into 12 major cell groups and 14 finely resolved subgroups (**Figure 2A**). Notably, the UMAP projection suggested pronounced LIMA1 expression specifically within malignant tumor cells and fibroblasts (**Figure 2B**). Comparative analysis of LIMA1 and RNF40 expression across all cell types further highlighted this cell-type specificity (**Figure 2C**). Quantification of cell-type proportions in LIMA1-positive versus LIMA1-negative populations revealed a significantly higher fraction of malignant cells in the LIMA1-positive group (**Figure 2D**). This expression pattern suggests a potential association between LIMA1 expression, malignant cell enrichment, and stromal components in the HCC microenvironment. Spatial transcriptomic profiling of four HCC specimens (LIHC1–LIHC4) supported spatial association between LIMA1 expression and tumor cell distribution (**Figure 2E**). LIMA1 was predominantly localized to tumor-enriched regions, with minimal detection in adjacent normal tissue. Delineation of malignant versus normal zones suggested significantly elevated LIMA1 expression in tumor regions (**Figure 2E**), corroborating single-cell findings.

### LIMA1 is associated with immune infiltration and checkpoint-related features in HCC

**Figure 3:**
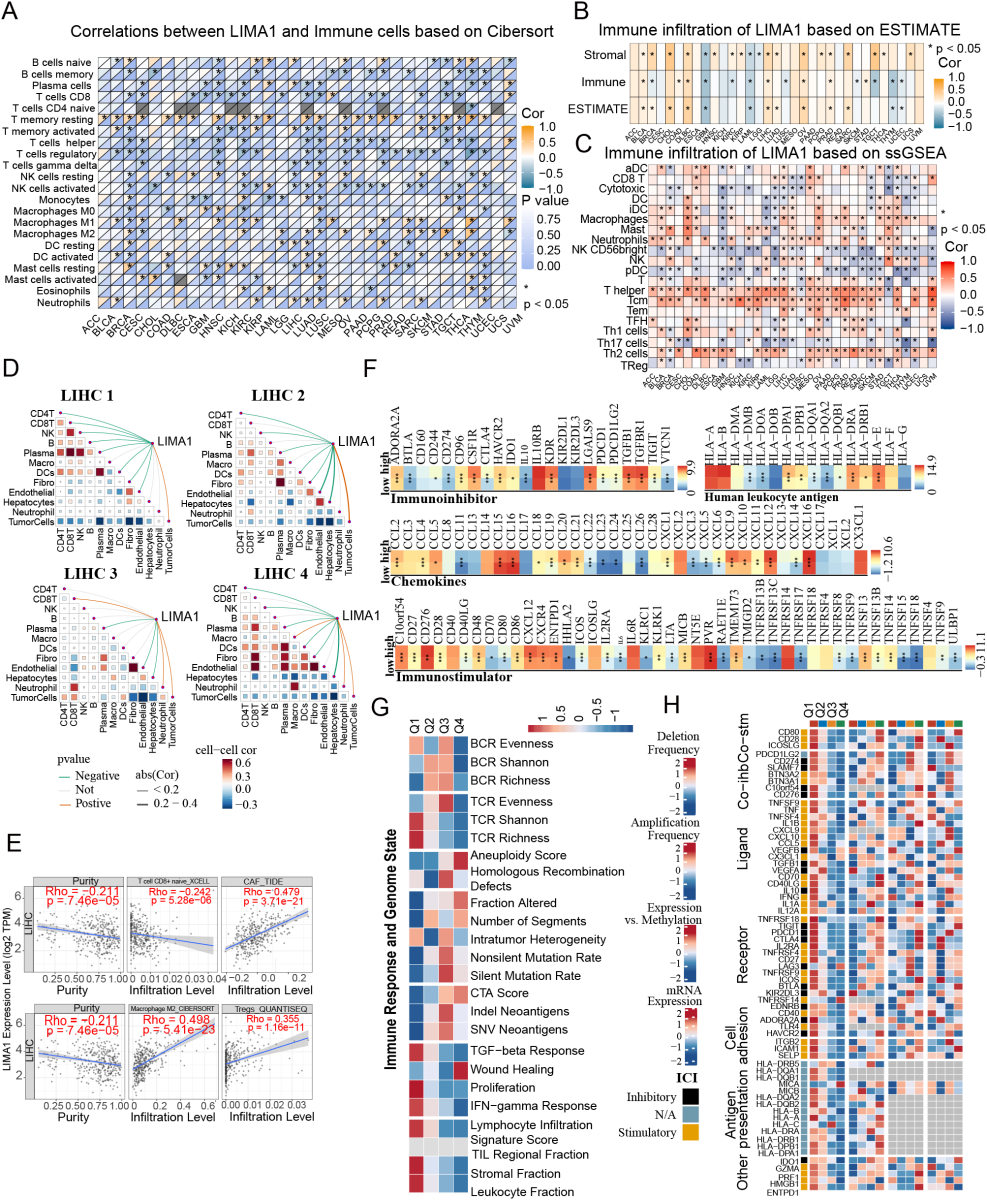
Association between LIMA1 expression and immune infiltration/checkpoint-related features in HCC. **(A-C)** Association between LIMA1 expression and immune cell infiltration using CIBERSORT, ESTIMATE, and ssGSEA algorithms. **(D)** Spearman correlation of LIMA1 expression with micro-environmental components at spatial resolution. **(E)** Differential expression landscape of LIMA1-associated immune regulators. **(F)** Correlation between LIMA1 and specific immune cell subsets via TIMER database analysis. **(G)** Relationship of LIMA1 expression with immune checkpoint activity and genomic instability. **(H)** Association between LIMA1 expression and immune checkpoint molecule levels.

Our comprehensive bulk transcriptomic profiling revealed significant correlations between LIMA1 expression levels and immune infiltration patterns through three established computational approaches: CIBERSORT for immune cell deconvolution (**Figure 3A**), ESTIMATE for microenvironment scoring (**Figure 3B**), and ssGSEA for pathway activity assessment (**Figure 3C**). Through spatial transcriptomic profiling, we quantitatively assessed the Spearman correlation between LIMA1 expression and tumor microenvironment components at a spatial resolution (**Figure 3D**). These findings demonstrated robust concordance with previous gene localization studies, revealing a statistically significant positive correlation between LIMA1 expression levels and malignant cell density within the tumor microenvironment. Notably, LIMA1 expression demonstrated modest but significant inverse correlations with key anti-tumor immune populations across distinct LIHC subtypes: LIHC1: CD4+ T cells, CD8+ T cells, NK cells, and B lymphocytes. LIHC2: CD4+ T cells, NK cells, and dendritic cells (DCs). LIHC3: Plasma cells. LIHC4: CD4+ T cells, NK cells, plasma cells, DCs, and tumor-associated macrophages. To comprehensively characterize LIMA1’s biological role, we performed integrated analyses evaluating its association with both immune microenvironment features (through immunogenicity scoring) and genomic instability markers (via DNA damage quantification), as presented (**Figure 3G**). Given the established role of immunomodulatory molecules in cancer immunotherapy, we systematically examined their correlation with LIMA1 expression patterns to establish an integrated profile of LIMA1-mediated immunoregulation (**Figures 3E, H**). Furthermore, we investigated correlations between LIMA1 expression and immune cell infiltration using the TIMER database (**Figure 3E**). Our analysis demonstrated significant positive correlations between LIMA1 expression levels and the abundance, (a) cancer-associated fibroblasts (R = 0.479, *P* = 3.71e-21), (b) M2 macrophages (R = 0.498, *P* = 5.41e-23), (c) regulatory T cells (Tregs) (R = 0.355, *P* = 1.16e-11). These cell types are established contributors to immunosuppressive tumor microenvironments (32). Conversely, LIMA1 expression showed a significant negative correlation with CD8^+^ naïve T cell infiltration (R = -0.242, *P* = 5.28e-06; **Figure 3F**). Collectively, these findings indicate that elevated LIMA1 expression is linked to an immunosuppressive tumor microenvironment. The potential role of RNF40-LIMA1 axis in immune cell infiltration was further analyzed (**Supplementary Figure 2**). We divided the HCC samples into two groups, LIMA1 high and RNF40 low and LIMA1 low and RNF40 high. The analysis found that there were significant differences in the infiltration levels of some immune cells between the two groups, which may provide clues for further study of the immune system. Additional ssGSEA-based immune infiltration and immune checkpoint correlation analyses further supported the association between LIMA1 and immune infiltration/checkpoint-related features (**Supplementary Figure 6**).

### Cell communication and hallmark functions of LIMA1 in HCC

**Figure 4:**
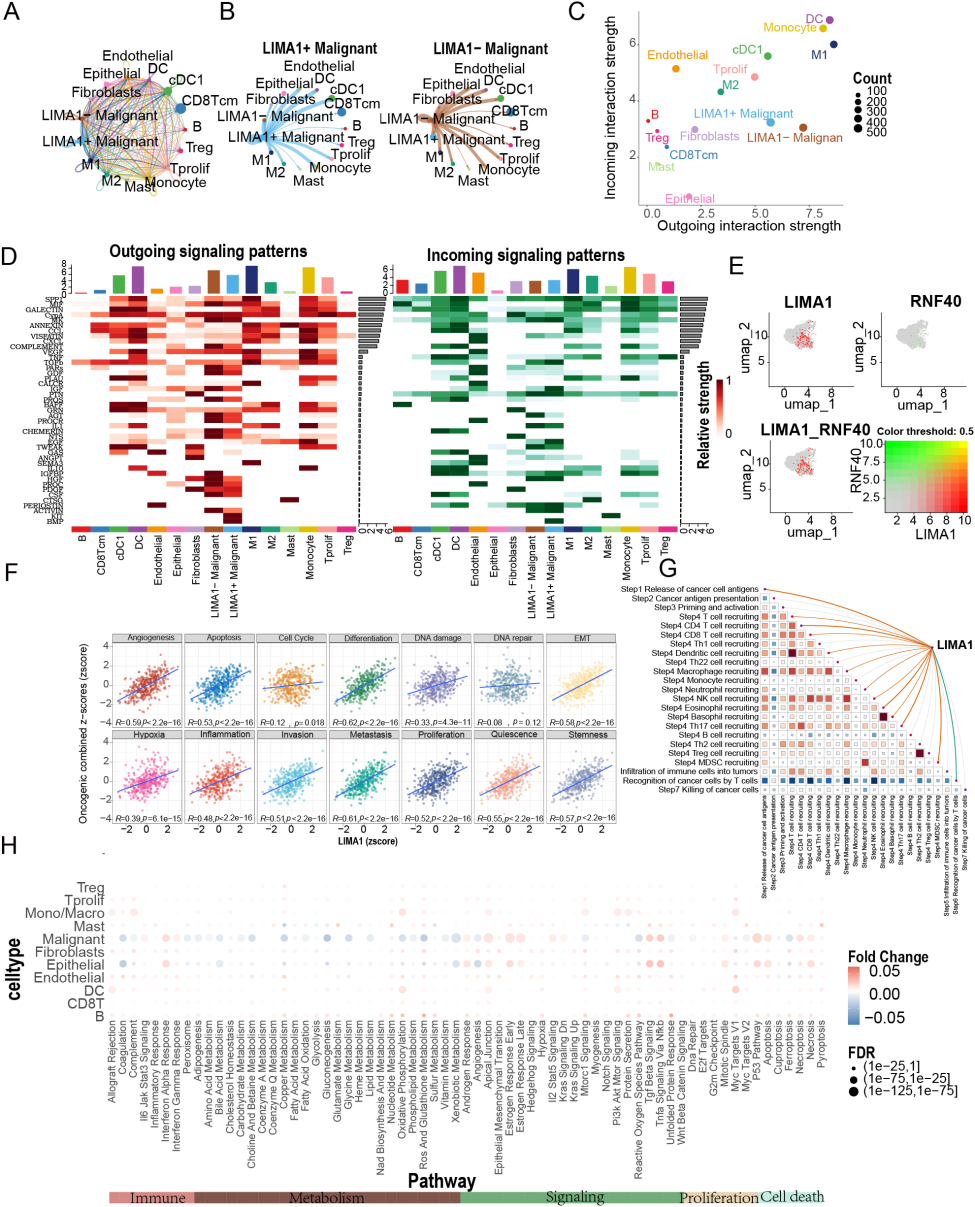
Cell communication and hallmark functions of LIMA1 in HCC. **(A)** Cell-cell interaction networks across distinct cell clusters. **(B)** Interaction landscapes of LIMA1^+^ vs. LIMA1^−^ malignant cells with other cell clusters. **(C)** Similarity analysis of interaction networks among cell clusters. **(D)** Top-weighted ligand-receptor pairs in each cluster. **(E)** Co-localization of LIMA1 and RNF40 in malignant cell clusters. **(F)** The correlation between cancer-related phenotypes and LIMA1 expression based on GSVA. **(G)** Spearman correlation between LIMA1 and TIP scores, as well as the autocorrelation between TIP scores. **(H)** The activation of some biological pathways among different cell clusters. *P < 0.05; **P < 0.01; ***P < 0.001.

Using CellChat, we mapped intercellular communication networks across cell lineages within the GSE166635 dataset (**Figure 4A**). We specifically delineated communication landscapes between LIMA1^+^ and LIMA1^-^ malignant cells relative to other clusters (**Figure 4B**). Comparative analysis revealed that while LIMA1^+^ and LIMA1^-^ malignant cells received signals at comparable levels, LIMA1^+^ malignant cells showed higher inferred outgoing signal activity than LIMA1^-^ malignant cells (**Figure 4C**), suggesting a potential association between LIMA1 expression and altered tumor-cell communication patterns. Further visualization highlighted the highest-weight signaling molecules driving differential communication patterns between clusters (**Figure 4D**). UMAP visualization showed co-expression patterns of LIMA1 and RNF40 within malignant cell populations (**Figure 4E**). Finally, we employed Gene Set Variation Analysis (GSVA) to evaluate correlations between LIMA1 expression and cancer-related phenotypic pathways. Our analysis demonstrated significant correlations between LIMA1 expression and multiple tumor phenotypes, angiogenesis (R = 0.59; *P* < 2.2e-16), apoptosis (R = 0.53; *P* < 2.2e-16), cell cycle (R = 0.12; *P* = 0.018), differentiation (R = 0.62; *P* < 2.2e-16), DNA damage (R = 0.33; *P* = 4.3e-11), EMT (R = 0.58; *P* < 2.2e-16), hypoxia (R = 0.39; *P* = 6.1e-15), inflammation (R = 0.48; *P* < 2.2e-16), invasion (R = 0.51; *P* < 2.2e-16), metastasis (R = 0.61; *P* < 2.2e-16), proliferation (R = 0.52; *P* < 2.2e-16), quiescence (R = 0.55; *P* < 2.2e-16), and stemness (R = 0.57; *P* < 2.2e-16) (**Figure 4F**). Notably, anti-cancer immunity operates through sequential biological events collectively termed the cancer immune cycle (33). We computed Spearman correlations between LIMA1 expression and Tumor Immune Proponent (TIP) scores, including TIP score autocorrelation, and visualized these relationships using the linkET package (**Figure 4G**). Furthermore, AUCell analysis revealed differential activation of key biological pathways across cell clusters, with malignant clusters exhibiting significant pathway regulation (**Figure 4H**).

### RNF40 inhibits the proliferation, migration and invasion of liver cancer cells via LIMA1

**Figure 5:**
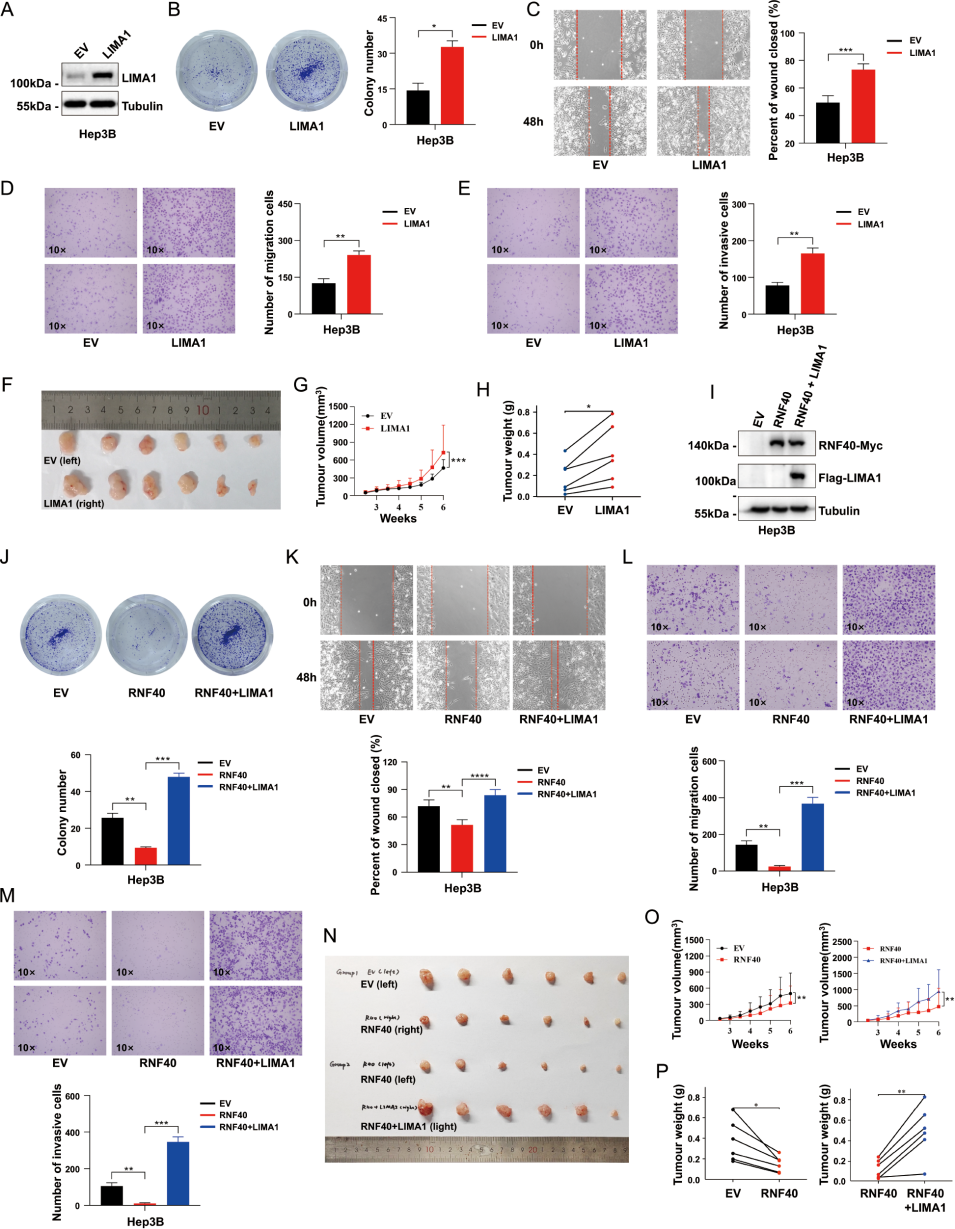
RNF40 suppresses malignant phenotypes of HCC cells in a LIMA1-dependent manner. **(A)** Protein expression levels of LIMA1. **(B)** Colony formation assay in Hep3B cells with LIMA1 overexpression. **(C-E)** Overexpression of LIMA1 promotes the migration, and invasion of Hep3B cells. **(F-H)** Tumor volume and weight in Hep3B xenografts grown in nude mice. **(I)** Protein expression levels of both LIMA1 and RNF40. **(J)** Assessing colony formation in Co-expression of LIMA1 and RNF40 Hep3B cells. **(K-M)** Co-expression of LIMA1 and RNF40 modulates the migration, and invasion of Hep3B cells. **(N-P)** Combined effects of LIMA1 and RNF40 on xenograft tumor volume and weight in nude mice. *P < 0.05; **P < 0.01; ***P < 0.001. ****P < 0.0001.

We generated stable LIMA1-overexpressing Hep3B cell lines. LIMA1 protein expression was significantly upregulated compared to empty vector (EV) controls, confirming successful overexpression (**Figure 5A**). Colony formation assays demonstrated enhanced clonogenic capacity in LIMA1-overexpressing cells, with quantitative analysis confirming increased colony numbers (**Figure 5B**). Concurrently, LIMA1 overexpression significantly enhanced Hep3B cell migration and invasion capabilities (**Figures 5C–E**), with quantitative analysis confirming increased wound closure and transwell migration. To evaluate LIMA1’s impact on tumor growth *in vivo*, we established xenograft models. Subcutaneous injection of LIMA1-overexpressing Hep3B cells markedly accelerated tumor progression compared to empty vector (EV) controls, as evidenced by increased tumor volume and weight (**Figures 5F–H**). Previous studies have reported that the E3 ubiquitin ligase RNF40 can specifically ubiquitinate LIMA1 and reduce its protein expression level (30).To evaluate the effect of E3 ubiquitin ligase RNF40 on HCC progression via LIMA1, we also established stable transfection cells with differential expression of RNF40 and LIMA1. Given that RNF40 promotes LIMA1 degradation through the ubiquitin-proteasome pathway, proteasome inhibitor MG132 (10 μM) was employed during the selection and maintenance phases to stabilize ectopically expressed LIMA1 protein in the RNF40+LIMA1 co-overexpression cells, as previously described (30). The Western blot results show that the stable transfection of RNF40 and RNF40+LIMA1 co-overexpression cells has been successfully established, with exogenous protein expression confirmed using tag antibodies and endogenous LIMA1 levels detected using an anti-LIMA1 antibody (**Figure 5I**). Notably, endogenous LIMA1 protein was downregulated upon RNF40 overexpression and upregulated following RNF40 CRISPR knockout, as previously demonstrated (30), confirming that RNF40 regulates both exogenous and endogenous LIMA1 protein levels. E3 ubiquitin ligase RNF40 significantly inhibited the clone formation, migration and invasion of cells, while overexpression of LIMA1 rescued this inhibition (**Figures 5J–M**). Subsequently, the *in vivo* effects of RNF40 were determined in nude mice inoculated with Hep3B cells. Six weeks inoculation later, we detected that administration of RNF40 significantly inhibits cancer growth when compared with mice treated with EV, with this inhibition rescued by LIMA1 overexpression as displayed in tumor volume and weight (**Figures 5N–P**). Together, these results support a functional antagonistic relationship between RNF40 and LIMA1 in HCC models.

## Discussion

Immune checkpoint inhibitors (ICIs) have emerged as a cornerstone of systemic HCC therapy in recent years, with regimens like atezolizumab/bevacizumab and tremelimumab/durvalumab now representing established first-line standards (34). However, intrinsic resistance affects 40-60% of advanced HCC patients, highlighting the critical need for predictive biomarkers and novel therapeutic targets (35, 36). LIMA1 exhibits context-dependent duality in oncology: while predominantly characterized as a tumor suppressor across multiple malignancies, emerging evidence paradoxically links it to oncogenic functions in head and neck squamous cell carcinomas (13, 14, 37–39). Recent studies suggest LIMA1 modulates cancer progression through key biological processes including actin dynamics, cell adhesion, metastasis, angiogenesis, and lipid metabolism (40–42). The role, regulatory mechanisms, and clinical significance of LIMA1 in hepatocellular carcinoma remain poorly defined. In this study, we comprehensively investigated LIMA1’s function in HCC using multi-omics approaches integrating transcriptomics, single-cell RNA sequencing, and spatial transcriptomics. Our analyses revealed consistent LIMA1 upregulation in HCC across multiple datasets, while its prognostic association varied across cohorts and endpoint settings. Single-cell resolution demonstrated predominant LIMA1 enrichment within tumor cell subpopulations, while spatial transcriptomics suggested tumor-enriched LIMA1 expression. These findings were validated through molecular biology experiments and animal studies, confirming that LIMA1 enhances HCC cell proliferation, migration, and invasive capacity.

Ubiquitination, a post-translational modification process—requires coordinated action of ubiquitin, ubiquitin-activating enzyme (E1), ubiquitin-conjugating enzyme (E2), and ubiquitin ligase (E3) to regulate protein stability, signaling pathway activation, and gene transcription. RNF40 (also termed BRE1B, RBP95, Staring, or KIAA0661) encodes a RING finger-type E3 ubiquitin ligase that monoubiquitinates histone H2B at lysine 120 (H2Bub1) (43). This enzyme epigenetically modulates target gene expression in a context-dependent manner (44). Significant knowledge gaps persist regarding cancer epigenome dynamics, gene regulation, and DNA repair mechanisms, driving growing interest in H2Bub1 and RNF40 functions during cancer-associated chromatin remodeling. Prior studies establish that RNF40 specifically ubiquitinates LIMA1, reducing its protein expression through proteasome-dependent degradation. This mechanism has been biochemically validated through co-immunoprecipitation, *in vivo* ubiquitination assays, cycloheximide chase experiments, and CRISPR-mediated knockout rescue experiments (30). While RNF40 exhibits tumor-suppressive activity in breast (45) and colorectal cancers (46), it paradoxically demonstrates oncogenic functions in prostate cancer (47), highlighting context-dependent roles in tumorigenesis. Although RNF40-mediated H2Bub1 participates in epigenetic regulation across malignancies, its mechanistic contributions to hepatocellular carcinoma pathogenesis remain poorly defined. We therefore systematically investigated the RNF40-LIMA1 functional interplay in HCC progression, revealing their coordinated regulation of tumor plasticity and microenvironment remodeling. Our experiments demonstrate that RNF40 significantly inhibits clonogenicity, migration, and invasion effects that were reversed by LIMA1 overexpression. Our research findings validated the differential expression of LIMA1 across multi-omics datasets, spatially resolved transcriptomics, and molecular assays. The current HCC experiments extend our previously established biochemical mechanism by showing that RNF40 suppresses malignant phenotypes in a LIMA1-dependent manner. However, several limitations should be acknowledged. First, this study was designed as a bioinformatics-driven investigation utilizing publicly available databases, and we did not have access to prospectively collected clinical samples for independent qPCR or IHC validation of LIMA1 and RNF40 expression. Future studies utilizing tissue microarrays or clinical biobank specimens would strengthen the biomarker claims. Second, the *in vitro* and *in vivo* functional experiments were performed exclusively in Hep3B cells. While CCLE database analysis confirmed RNF40-LIMA1 co-expression across 15 diverse HCC cell lines, functional validation in additional HCC models (e.g., Huh7, PLC/PRF/5) and LIMA1 loss-of-function experiments would enhance the generalizability of our conclusions. Third, the single-cell RNA sequencing analysis was based on a limited number of HCC patients (n = 2 in GSE166635), which may affect the robustness of conclusions regarding cell-type-specific LIMA1 distribution. The use of larger scRNA-seq cohorts (e.g., GSE125449, n = 19) would address this concern. Fourth, the GSE215011 immunotherapy cohort was small (n = 10), precluding definitive conclusions about LIMA1 as an immunotherapy response predictor. Fifth, while our companion study (30) established the biochemical mechanism of RNF40-mediated LIMA1 ubiquitination, the specific lysine residues on LIMA1 targeted by RNF40 were not identified and warrant further investigation. Finally, IHC staining of xenograft tumor tissues for LIMA1, RNF40, and immune cell infiltration was not performed due to sample availability constraints. A summary of the previously published biochemical evidence supporting RNF40-mediated LIMA1 ubiquitination and proteasome-dependent degradation is provided for context in **Supplementary Figure 8**.

We initially hypothesized that LIMA1 may serve as a companion biomarker for immunotherapy response prediction based on its significant correlations with immune checkpoint molecule expression (PD-L1, TIM-3, TIGIT, PD-1, CTLA-4) and immunosuppressive microenvironment features. To explore this hypothesis, we searched for publicly available HCC immunotherapy cohorts with gene expression and treatment response data. Analysis of the GSE215011 nivolumab-treated HCC cohort (n = 10) revealed numerically higher LIMA1 expression in non-responders compared to responders (Cohen’s d = 0.944, large effect size; ROC AUC = 0.72), although statistical significance was not reached due to the limited sample size (Wilcoxon P = 0.25). Mechanistically, LIMA1 has been established as a negative regulator of Wnt/β-catenin signaling, and Wnt/β-catenin pathway activation is a well-documented driver of immune exclusion and resistance to immune checkpoint inhibitors in HCC (Ruiz de Galarreta et al., Cancer Discov, 2019; Spranger et al., Nature, 2015). Although LIMA1 expression showed positive correlations with several checkpoint genes and a non-significant trend in a small ICI-treated HCC cohort, these observations are insufficient to support LIMA1 as a predictive biomarker for immunotherapy response. Therefore, we present this result as a hypothesis-generating finding only (**Supplementary Figure 7**).

Although our data support a functional RNF40-LIMA1 axis in HCC, several conclusions should be interpreted cautiously. First, LIMA1 did not remain an independent prognostic factor in our re-analysis after adjustment for available clinicopathological variables. Second, immune checkpoint correlations and the small GSE215011 cohort support only a hypothesis-generating link between LIMA1 and immunotherapy response. Third, although RNF40-mediated LIMA1 ubiquitination and proteasomal degradation were established in our previous work, further biochemical validation in additional HCC models and identification of LIMA1 ubiquitination sites are needed.

## Conclusions

In summary, our study shows that LIMA1 is upregulated in HCC and is associated with malignant phenotypes and immune microenvironment features. Functional experiments indicate that LIMA1 promotes HCC cell proliferation, migration, invasion, and xenograft growth, whereas RNF40 suppresses these malignant phenotypes in a LIMA1-dependent manner. Together with previously established biochemical evidence of RNF40-mediated LIMA1 ubiquitination and proteasome-dependent degradation, our findings support the biological relevance of the RNF40-LIMA1 axis in HCC progression. Further validation in independent clinical cohorts, additional HCC cell models, and larger ICI-treated cohorts is required to define its prognostic and therapeutic relevance.

## Materials and methods

### Dataset collection and preprocessing

In this investigation, the TCGA-LIHC datasets were employed, consisting of a total of 423 samples, including 50 normal tissues and 373 tumor specimens. This dataset incorporated RNA sequencing data along with clinical information retrieved from the TCGA database (https://portal.gdc.cancer.gov/). Patients who lacked survival data within the TCGA-LIHC cohort were excluded from the analysis. For subsequent analyses, Ensemble IDs were converted into gene symbols. Furthermore, additional datasets were sourced from the GEO database (https://www.ncbi.nlm.nih.gov/geo/), specifically the GSE39791 dataset, which includes 72 tumor samples and adjacent tissues; the GSE14520 dataset, comprising 245 tumor samples alongside 239 normal tissues; and the GSE112790 dataset, which contains 182 tumor samples and 15 normal tissues. Proteomic data concerning hepatocellular carcinoma were obtained from PDC000198 within the National Cancer Institute’s Proteomic Data (https://proteomic.datacommons.cancer.gov/pdc/). For the single-cell analysis, the GSE166635 dataset was downloaded from the GEO database, encompassing 25,189 cells sourced from two patients diagnosed with HCC (https://www.ncbi.nlm.nih.gov/geo/). The preprocessing procedures included quality control, cell clustering, differential expression analysis, cell type annotation, and classification of malignant cells, as outlined in the TISCH workflow (15).

Spatial transcriptome profiles were obtained from PMID: 36708811 and are available in the Mendeley Database (identifier: skrx2fz79n). This dataset comprises four HCC samples, labeled LIHC1, LIHC2, LIHC3, and LIHC4, which correspond to P7T, P9T, P10T, and P15T in the Mendeley database, respectively (16). All datasets analyzed in this study are readily accessible through previous publications and various public databases.

### Bulk transcriptomic analysis

Patients were categorized based on the median expression level of LIMA1 across all cohorts. Differential expression analysis was conducted across the TCGA-LIHC, GSE14520, GSE39791, and GSE112790 cohorts, incorporating both paired and unpaired analyses within the TCGA cohort. Additionally, differential analysis was performed within the combined TCGA and GTEx cohort (17).

Survival analysis was carried out to compare groups with high and low expression of LIMA1. In the TCGA cohort, Kaplan-Meier curves were generated utilizing the ‘survminer’ and ‘survival’ packages to investigate the association between LIMA1 expression and prognosis in HCC. An analysis of LIMA1 protein levels was conducted based on the PDC000198 cohort.

The differential expression analysis was performed using the ‘limma’ package, applying thresholds of log fold change (logFC) greater than 1.0 and a p-value of less than 0.001 to identify significant differences between the two groups, resulting in a list of differentially expressed genes (18). These genes were subsequently utilized in KEGG and GSEA enrichment analyses.

For bulk transcriptome analysis, immune infiltration was assessed using algorithms such as ssGSEA, XCELL, and CIBERSORT (19–21). We quantified the correlation between LIMA1 expression and the immune microenvironment, which included immune cells, immune molecules, and immune response states within the TCGA cohort.

Patients were divided into four groups based on gene expression quartiles: Q1, Q2, Q3, and Q4. Q1 represented the top 25% of samples with the highest LIMA1 expression, while Q4 encompassed the bottom 25% with the lowest expression. Following the methodology established by Thorsson V et al. regarding immune response and genomic status, we calculated the average score for each group, excluding missing values, and visualized the results using the ‘pheatmap’ package (22).

The CancerSEA database was utilized to classify the various functional states of 14 tumor cell types (23). The activity of specific pathways was assessed by integrating the expression profiles of characteristic genes. Using the R package ‘GSVA’ with the z-score parameter, we calculated the gene sets corresponding to the 14 functional states and obtained a combined z-score. This score was then standardized as the gene set score, and we computed the Pearson correlation between LIMA1 expression and each of the gene set scores.

### Multivariable prognostic modeling

To evaluate LIMA1 as an independent prognostic factor, multivariable Cox proportional hazards regression was performed in the TCGA-LIHC cohort using the ‘survival’ and ‘survminer’ packages in R. The model included LIMA1 expression (dichotomized by median split and as a continuous variable) along with clinically relevant covariates: tumor stage (I/II/III/IV), histological grade (G1/G2/G3/G4), vascular invasion (present/absent), AFP level (elevated/normal), age (continuous), and sex. Hazard ratios (HR) with 95% confidence intervals and P-values were calculated. The proportional hazards assumption was tested using Schoenfeld residual analysis (‘survival’ package). Time-dependent ROC curves for 1-year, 3-year, and 5-year OS and DFI prediction were generated using the ‘timeROC’ package, comparing three models: (i) LIMA1 alone, (ii) clinical variables alone, and (iii) LIMA1 combined with clinical variables. Decision curve analysis (DCA) was performed using the ‘rmda’ package to assess clinical net benefit. Calibration curves comparing predicted vs. observed survival probabilities were constructed, and a nomogram integrating LIMA1 with clinical variables was developed using the ‘rms’ package for individualized risk prediction. The primary multivariable model included LIMA1 expression and clinicopathological variables with relatively complete annotations, including age, pathological stage, and histological grade. Variables with substantial missingness, such as AFP level and vascular invasion, were evaluated descriptively but were not included in the primary model.

### RNF40-LIMA1 expression correlation analysis

Pearson and Spearman correlation analyses between RNF40 and LIMA1 mRNA expression were performed in tumor tissues across the TCGA-LIHC, GSE14520, GSE39791, and GSE112790 cohorts. Differential expression of RNF40 between tumor and adjacent normal tissues was evaluated using the Wilcoxon rank-sum test. Additionally, RNF40 and LIMA1 expression data for 15 HCC cell lines were extracted from the Cancer Cell Line Encyclopedia (CCLE) database, and Pearson correlation was calculated.

### Single-cell RNA sequencing (scRNA-seq) data processing and analysis

Single-cell data processing was performed using the R software package “Seurat” (24). Cells that exhibited gene expression levels between 500 and 8000, as well as those with mitochondrial gene expression surpassing 15%, were excluded from the dataset. The gene expression values were then normalized using the SCTransform method, followed by dimensionality reduction via principal component analysis (PCA). To address inter-sample batch effects, the “Harmony” package was employed. Clustering analyses were conducted using the Seurat functions (FindNeighbors and FindClusters) (25), and the outcomes of these analyses were visualized using Uniform Manifold Approximation and Projection (UMAP). For cell type identification, we referred to the CellMarker database (http://xteam.xbio.top/CellMarker/index.jsp) to obtain liver tissue-specific cell marker gene information, enhancing the accuracy of our cell type classifications (26).

The UMAP technique was used to visualize the expression levels of LIMA1 and RNF40. To assess differences in the expression of these genes across various cell types, we applied the Kruskal-Wallis rank sum test. To understand the distribution of LIMA1 across all cell types, cells were categorized into LIMA1-positive and LIMA1-negative groups based on their expression levels. Specifically, LIMA1-positive and LIMA1-negative malignant cells were classified based on median expression cutoff (≥ median value defined as positive, < median as negative). The proportion of each cell type within these positive and negative groups was subsequently calculated.

To explore the functional pathway heterogeneity between these two subgroups, we conducted enrichment analysis and cell communication analysis. The AUCell package was utilized to evaluate scores associated with immune, metabolic, and signaling pathways, as well as biological pathways related to proliferation and cell death. Cells were classified into LIMA1-positive and LIMA1-negative groups according to their LIMA1 expression levels, and the limma package was employed to analyze the differences in scores between these groups. In cases where either the positive or negative group contained zero cells, those cells were excluded from further analyses.

Furthermore, the CellChat package (version 1.6.1) was applied to analyze and visualize intercellular communication, considering gene expression data alongside known interactions among signal transduction ligands, receptors, and cofactors (27). Malignant tumor cells were divided into two groups based on LIMA1 expression levels, and differences between these groups were examined through cell communication analysis. Finally, we extracted the subgroup of malignant cells to evaluate the co-expression of RNF40 alongside LIMA1.

### Spatial transcriptomic analysis

Spatial transcriptome analysis offers insights into the spatial distribution and functional role of LIMA1 within the tumor microenvironment (TME) (28, 29) To accurately evaluate the cellular composition of each spot on the 10x Visium slides, we utilized the ‘SPOTlight’ package to conduct a deconvolution analysis. Stringent quality control measures were implemented to ensure the reliability of our results, focusing on the number of expressed genes, the count of unique molecular identifiers (UMIs), and the percentage of mitochondrial RNA present in each cell.

Following this, we computed the average expression of the top 25 specifically expressed genes for each cell type in the scRNA-seq reference for each locus, thereby creating a signature score matrix. Subsequently, we employed the get_enrichment_matrix and enrichment_analysis functions from the ‘Cottrazm’ package to generate an enrichment scoring matrix, which provided robust support for the subsequent analysis of cellular composition. The SpatialFeaturePlot function in the Seurat package was then used to visualize the enrichment scores of each cell type, with higher scores depicted in darker colors, indicating a greater abundance of that cell type within each spot.

We defined the malignant group as having a score of 1 for malignant cells in the microregion, the normal group as having a score of 0, and the mixed group as having intermediate scores. To assess the significance of statistical differences in the expression of specific genes among the three subgroups, we applied Wilcoxon rank sum tests in a pairwise manner.

Utilizing the results from the deconvolution analysis, we identified the cell type with the highest abundance in each microregion and visualized the maximum cellular component value in each microregion using the SpatialDimPlot function from the ‘Seurat’ package. Additionally, the SpatialFeaturePlot function was employed to illustrate the expression landscape of LIMA1 across each microregion. To examine correlations between cell abundances and LIMA1 expression across all spots, we conducted Spearman correlation analysis, visualizing the results with the ‘linkET’ package.

### Cell lines

The liver cancer cell lines were gifts from Zhang lab at The Institute of Molecular and Translational Medicine, Xi’an Jiaotong University. Establishment of RNF40+LIMA1 co-overexpression cells. To establish stable Hep3B cells co-expressing RNF40 and LIMA1, cells were sequentially transduced with lentiviral particles carrying pLVX-RNF40-Myc and pLVX-LIMA1-Flag. During the puromycin selection phase and subsequent maintenance, proteasome inhibitor MG132 (10 μM; MCE, HY-13259) was added to the culture medium to prevent RNF40-mediated degradation of ectopically expressed LIMA1 protein. This approach is based on our previous demonstration that MG132 completely blocks RNF40-mediated LIMA1 degradation (30). MG132 was withdrawn 24 hours prior to functional assays to minimize off-target effects.

### Reagents

Lipofectamine 3000 reagent (Thermo Fisher Scientific), Puromycin (Thermo Fisher Scientific, A1113803), Polybrene (Beyotime Biotechnology, C0351), Crystal Violet Staining Solution (Beyotime Biotechnology, C0121).

### Western blot analysis

Protein levels were determined by immunoblotting as previously described (31). Briefly, cultured cells were lysed with NP40 buffer containing protease inhibitors. Western blots were obtained utilizing 20-40μg of lysate protein. The following antibodies were used in this study: anti-Beta Tubulin antibody (Proteintech, 66240, 1:10,000 dilution), LIMA1 rabbit mAb (Thermo Fisher Scientific, PA5-31567, 1:1000 dilution), anti-Flag and anti-Myc tag rabbit mAb (Cell Signaling Technology, 14793S, 2278S, 1:1000 dilution).

### Colony formation assay

Colonies were quantified by manual counting of cells in four randomly selected peripheral fields per well, avoiding central regions exhibiting non-uniform cell distribution. This approach ensured standardized evaluation across all experimental conditions.

### Cell scratch assay

Hep3B cells were collected and made into cell suspensions, which were seeded on a six-well plate (at about 6×10^5^ cells/well) to ensure that the cells could spread over the entire well overnight. The monolayer was scratched across the center of each well utilizing the tip of a 200 µL pipette, washed the 6-well plate with PBS to wash away the free cells, added serum-free medium for cells to culture, and take pictures under the microscope at 0, 12, and 24 h after scratching to record the scratch diameter of each well. Each scratch wound was visualized by microscopy and five random fields (×100) were chosen to assess the cell migration ability.

### Transwell

Traditional Transwell assays were performed in a Corning HTS Transwell 96-well plate containing polycarbonate membranes with 8 μm-diameter pores. After 12 h of serum-free starvation, Hep3B cells in the logarithmic growth phase were harvested using trypsin-EDTA, and resuspended in serum-free medium, and adjusted to a cell density of 1×10^6^ cells/mL. Cells in 200 µL of serum-free medium were seeded on the upper chamber, and 600 µL of medium supplemented with 10% FBS was placed in the lower chamber. After 24 h of incubation, the chamber was fixed with 4% paraformaldehyde and subsequently dyed with 0.1% crystal violet. The upper chamber was carefully wiped with cotton swabs. The numbers of cells that had migrated to the lower surface were enumerated in five randomly selected visual fields (×200). For the cell invasion assay, prior to cell inoculation, the chamber pores were pre-filled with a mixture of serum-free culture medium containing matrix gel and incubated overnight at 37°C. Afterward, cells can be inoculated, and the subsequent experimental methods are the same as those for the cell migration assay.

### Xenograft model

The xenograft experiments were performed in accordance with the National Guidelines for Experimental Animal Welfare and with approval of the Ethics Committee of Xi’an Jiaotong University (Approval No. XJTUAE2023-1500). 4 to 6-week-old balb/c nude mice were purchased from Beijing Vi-tal River Laboratory Animal Technology Co, Ltd., and were used for cell line xenograft experiments. The Hep3B cells were resuspended in the FBS-free DMEM medium and were injected into mice subcutaneously. The tumor volume was calculated according to the equation: volume = 0.5× length × width^2^. Animals were sacrificed when the average tumor volume reached 1000 mm^3^.

### Statistical analysis

Data are expressed as Mean ± S.E.M. Normality was assessed using the Shapiro-Wilk test. Sample sizes (n) are reported for all analyses, including the number of patients per cohort, the number of cells per cluster, and the number of biological replicates for *in vitro* and *in vivo* experiments. Group differences were evaluated using Student’s t-test for two-group comparisons or one-way ANOVA followed by Tukey’s *post hoc* test for multiple comparisons, utilizing GraphPad Prism 6 (GraphPad Software, Inc., La Jolla, CA). A p-value of less than 0.05 was deemed statistically significant.

## Data Availability

The datasets presented in this study can be found in online repositories. The names of the repository/repositories and accession number(s) can be found in the article/**Supplementary Material**.
