# Supplementary figures and images for: Integrative multi-omics analysis implicates the RNF40-LIMA1 axis in hepatocellular carcinoma progression and immune microenvironment remodeling
Source: Front Oncol. 2026 Jun 29;16:1846606. doi: 10.3389/fonc.2026.1846606 (PMC13357399; doi:10.3389/fonc.2026.1846606)

Figure S1

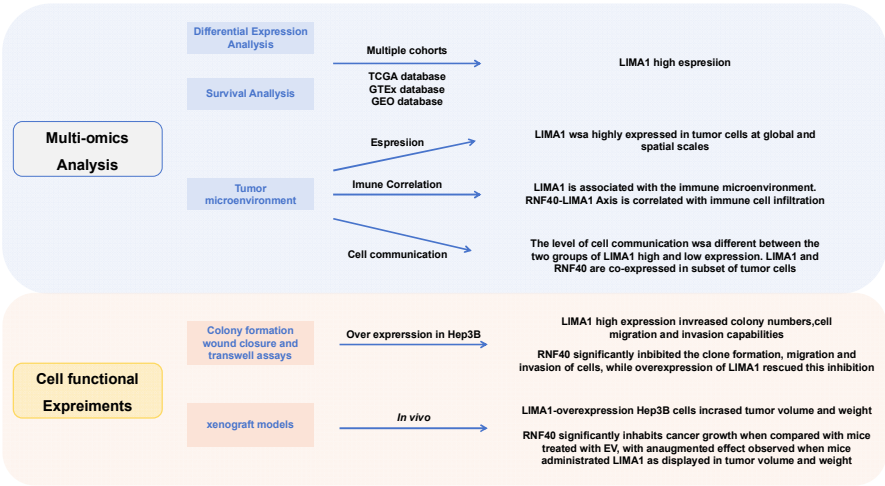

Figure S2

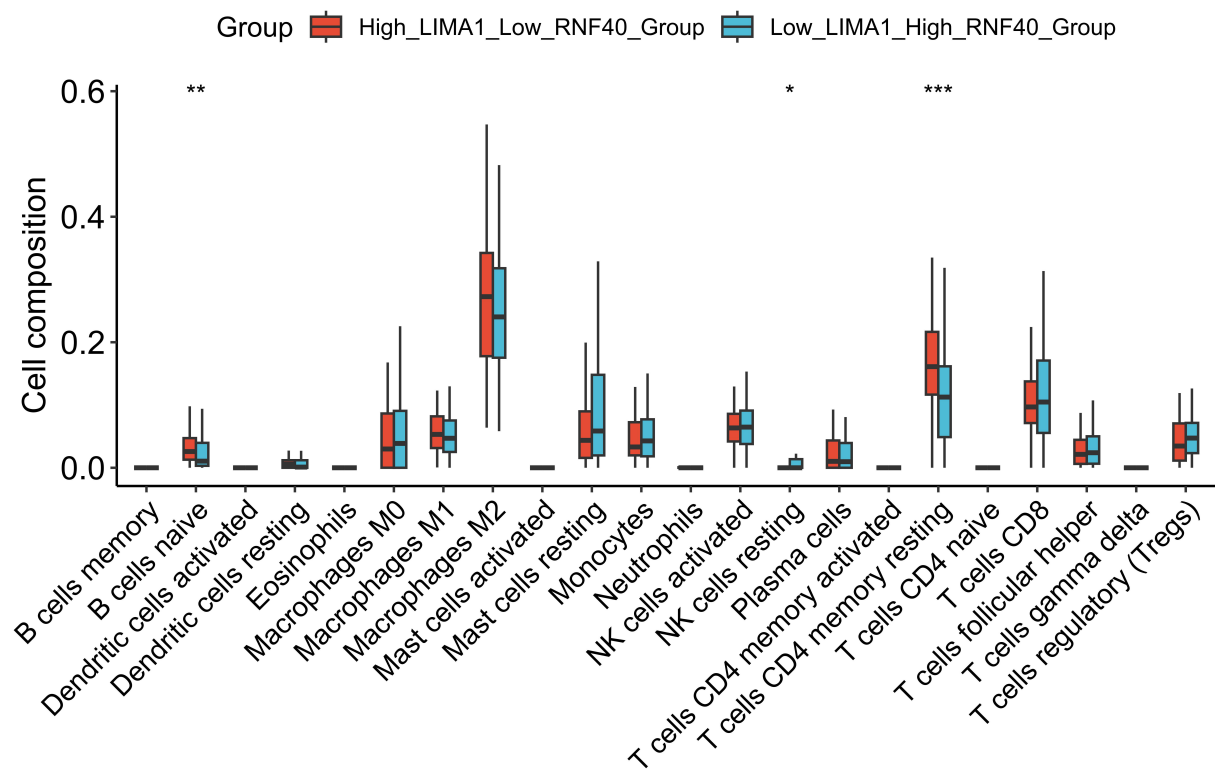

Figure S3

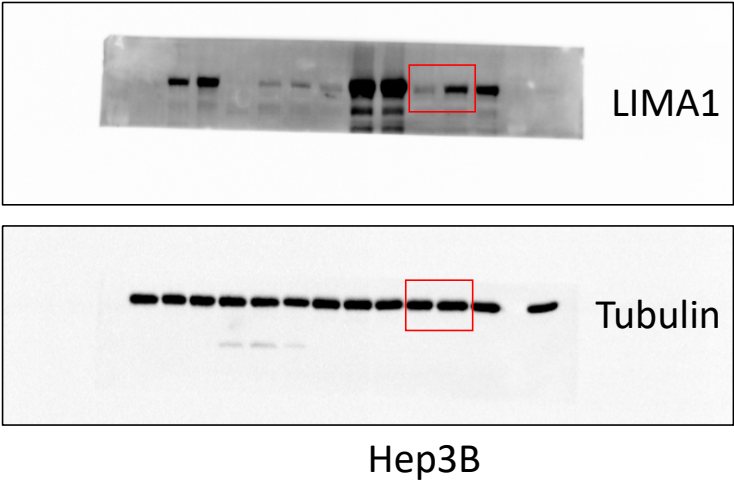

fig.5 A

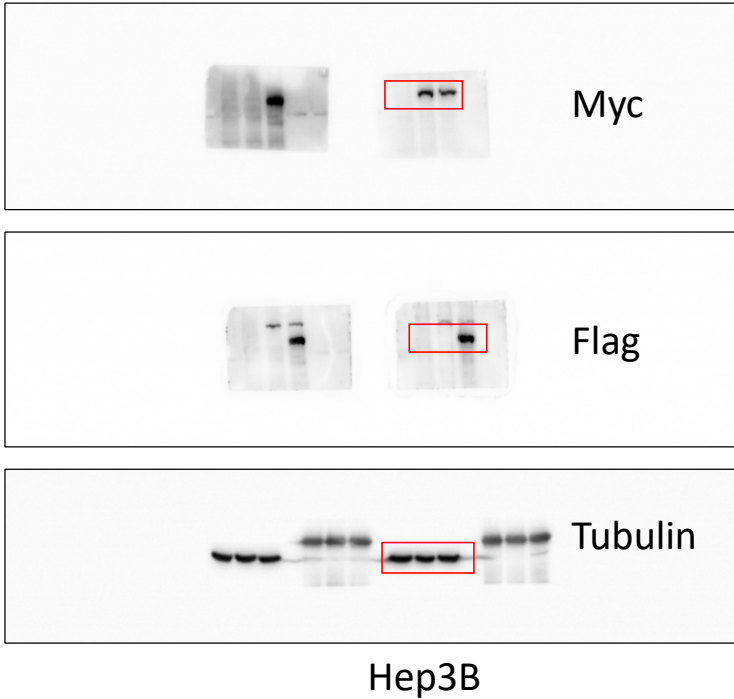

fig.5 I

Supplement: Supplementary file 1 [file DataSheet1.pdf]

Figure S4.

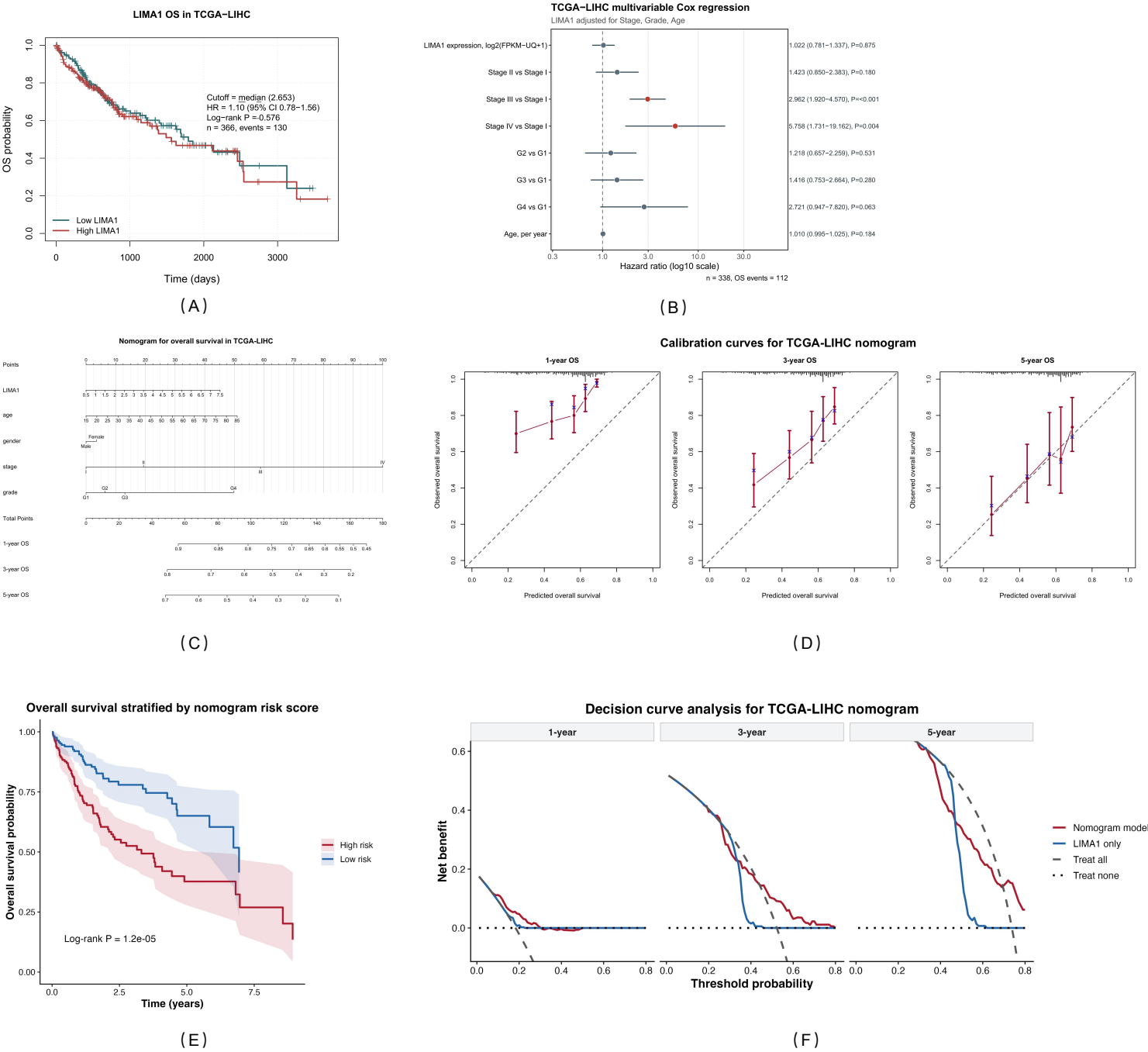

Figure S5.

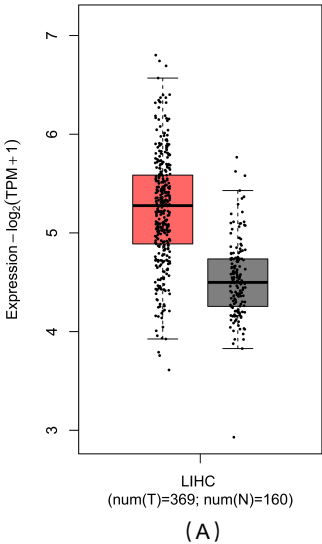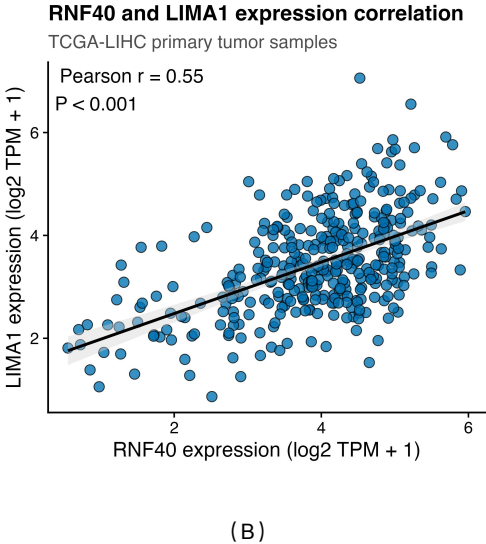

Figure S6.

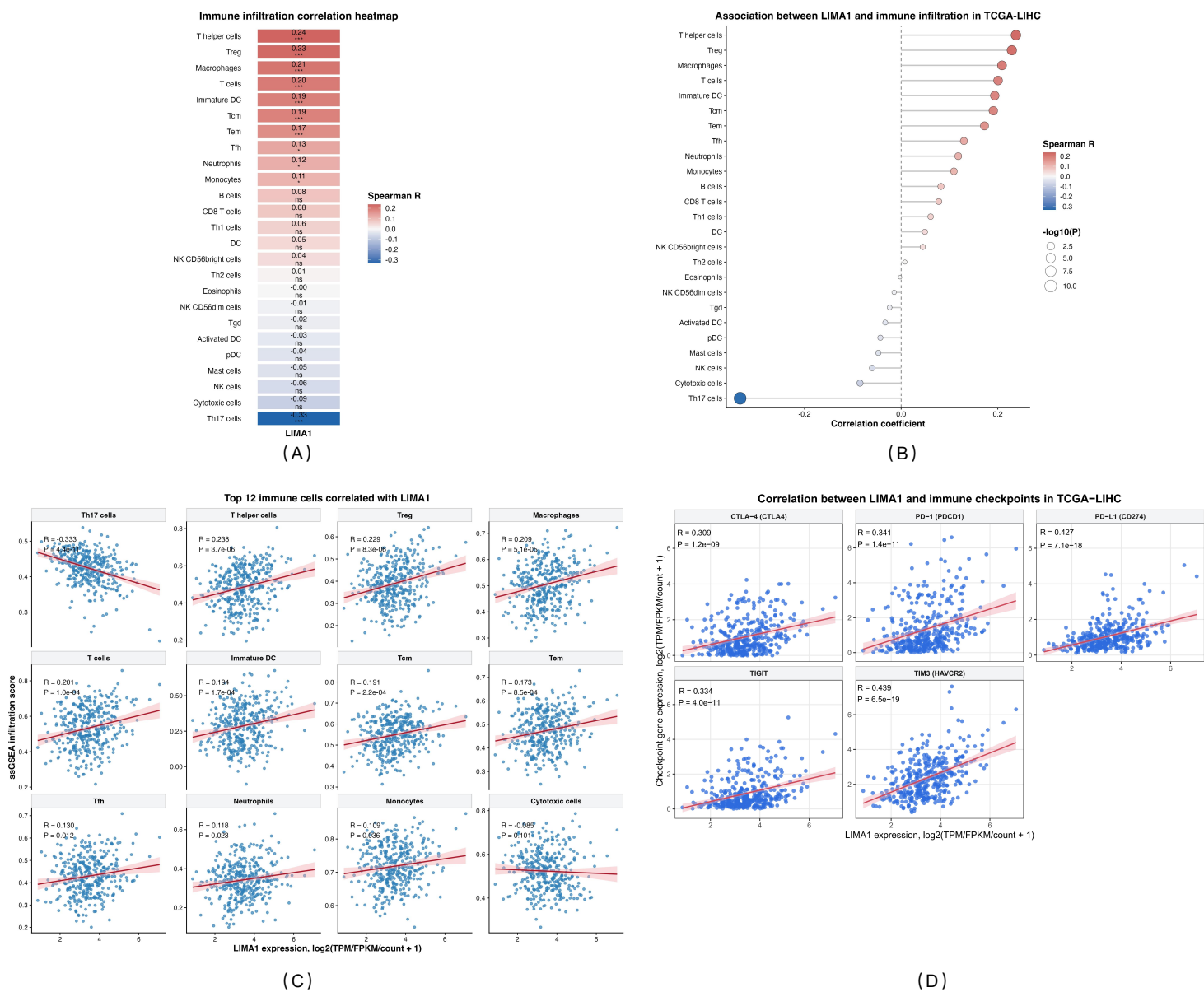

Figure S7.

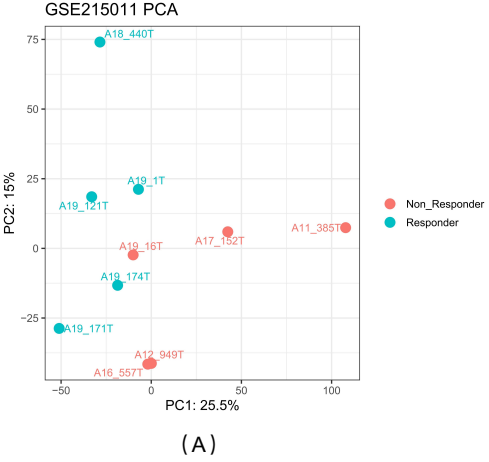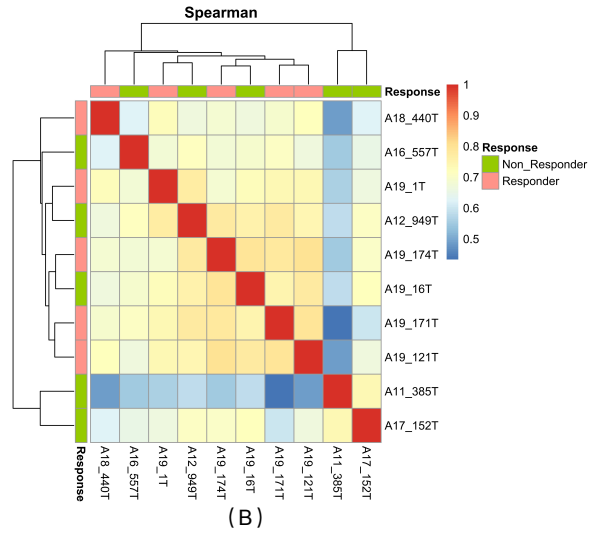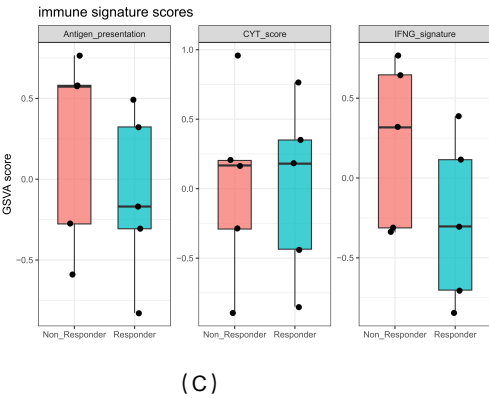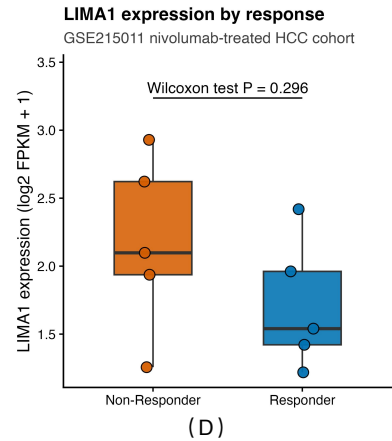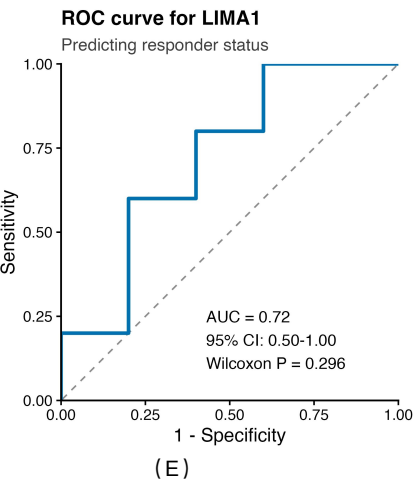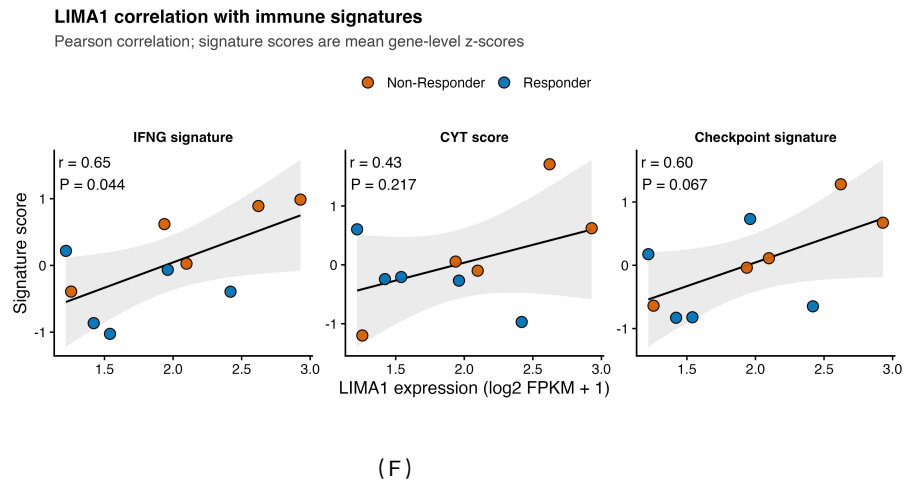

Figure S8.

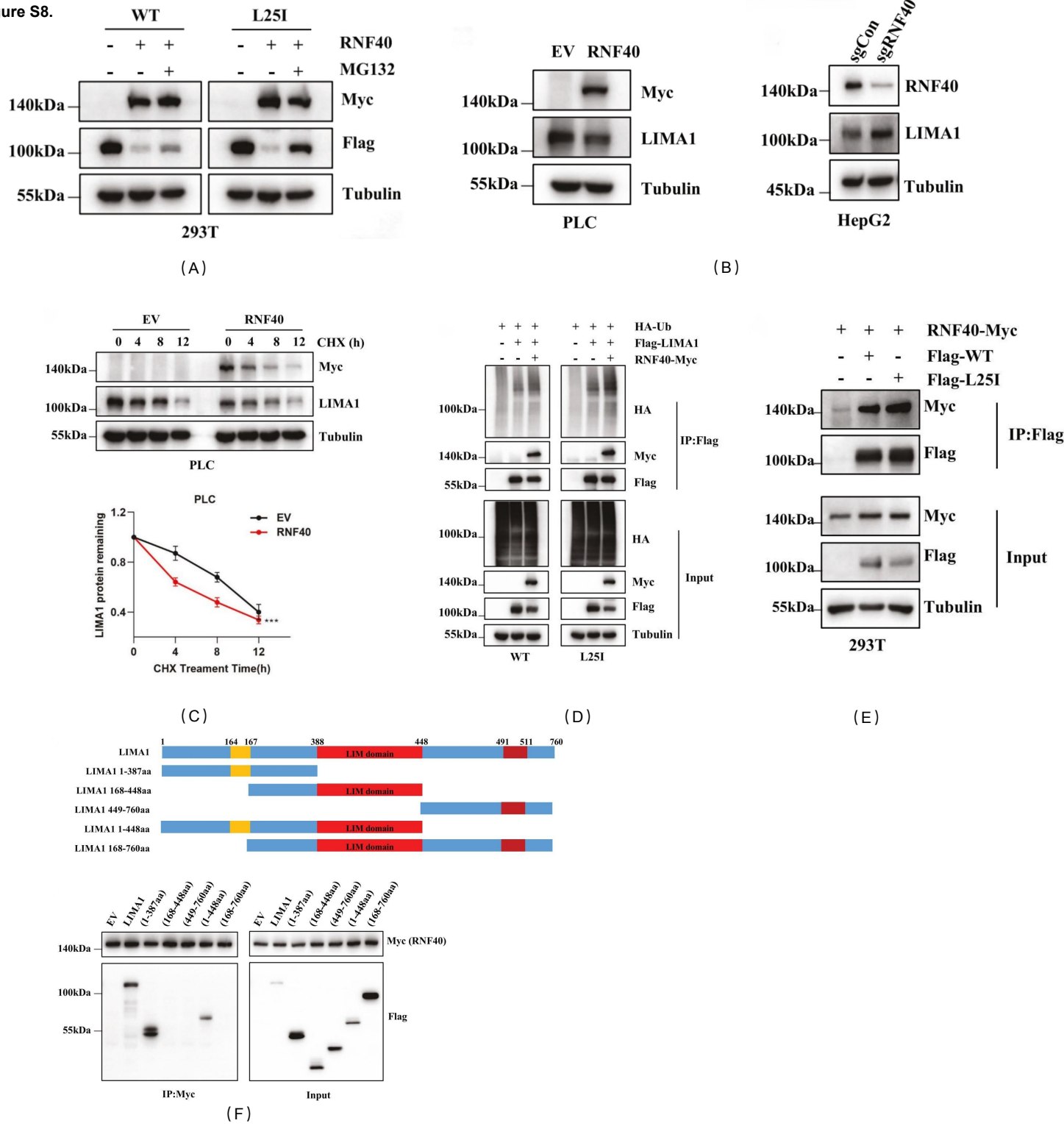

Supplement: Supplementary file 3 [file DataSheet3.pdf]
