# Supplementary material for: Integrative multi-omics analysis implicates the RNF40-LIMA1 axis in hepatocellular carcinoma progression and immune microenvironment remodeling
Source: Front Oncol. 2026 Jun 29;16:1846606. doi: 10.3389/fonc.2026.1846606 (PMC13357399; doi:10.3389/fonc.2026.1846606)
Supplement: Supplementary file 2 [file DataSheet2.pdf]

Supplementary Figure Legends

Figure S4：Re-analysis of prognostic value and nomogram:(A) TCGA-LIHC LIMA1 median cutoff KM for OS (HR=1.10，P=0.576).(B) Multivariable Cox forest plot.(C) Nomogram integrating LIMA1 and clinicopathological variables.(D) Calibration curves for 1-, 3-, 5-year OS.(E) DCA for 1-, 3-, 5-year OS.(F) Nomogram risk score stratified KM.

Figure S5：RNF40 expression and RNF40-LIMA1 correlation:(A) RNF40 expression in LIHC tumor and normal tissues (tumor n=369,normal n=160).(B) RNF40-LIMA1 mRNA correlation (R=0.28，P=7e-08).

Figure S6：Immune infiltration and checkpoint correlations:(A) Immune infiltration heatmap.(B) Lollipop plot.(C) Top immune cell scatter plots.(D) Immune checkpoint scatter plots：CTLA4、PDCD1、CD274、TIGIT、HAVCR2.

Figure S7：GSE215011 exploratory immunotherapy-response analysis:(A) PCA of responder vs non-responder，PC1 = 25.5%，PC2 = 15%.(B) Top 50 DEG heatmap.(C) Antigen presentation / CYT score / IFNG signature.(D) LIMA1 expression in responder vs non-responder.(E) ROC curve for LIMA1 predicting response.(F) LIMA1 correlation with IFNG/CYT/checkpoint signatures.

Figure S8：Previously published biochemical evidence supporting RNF40-mediated LIMA1 ubiquitination and proteasome-dependent degradation.
(A) Proteasome inhibition by MG132 restored RNF40-induced reduction of LIMA1 protein levels, adapted from Fig. 2C of Liu et al., Cell Death Discovery, 2024.
(B) RNF40 overexpression decreased endogenous LIMA1 protein levels, whereas RNF40 knockout increased endogenous LIMA1 protein levels, adapted from Fig. 2D–E.
(C) CHX chase assay showing that RNF40 overexpression decreased LIMA1 protein stability, adapted from Fig. 2F–G.
(D) RNF40 increased polyubiquitination of LIMA1-WT and LIMA1-L25I, adapted from Fig. 2H.
(E) Co-immunoprecipitation showing the interaction between RNF40 and LIMA1, adapted from Fig. 3A and/or Fig. 3C.
(F) Domain-mapping analysis showing that the N-terminal 1–166 aa region of LIMA1 is required for interaction with RNF40, adapted from Fig. 3D–E.
Panels were reproduced/adapted from Liu et al., Cell Death Discovery, 2024, under the applicable license.
