# Supplementary material for: Integrative multi-omics analysis implicates the RNF40-LIMA1 axis in hepatocellular carcinoma progression and immune microenvironment remodeling
Source: Front Oncol. 2026 Jun 29;16:1846606. doi: 10.3389/fonc.2026.1846606 (PMC13357399; doi:10.3389/fonc.2026.1846606)
Supplement: Supplementary file 4 [file Table1.docx]

**Supplementary Figure Legends**

**Figure S1.** The flowchart of this study.

**Figure S2.** Differences in immune cell infiltration levels between LIMA1 high expression RNF40 low expression group and LIMA1 low expression RNF40 group in TCGA-LIHC samples.

**Figure S3.** Original, uncropped Western blot images. Red boxes denote the cropped regions presented in main figures.
